# Parthenolide attenuated bleomycin-induced pulmonary fibrosis via the NF-κB/Snail signaling pathway

**DOI:** 10.1186/s12931-018-0806-z

**Published:** 2018-06-05

**Authors:** Xiao-he Li, Ting Xiao, Jia-huan Yang, Yuan Qin, Jing-jing Gao, Hui-juan Liu, Hong-gang Zhou

**Affiliations:** 1 State Key Laboratory of Medicinal Chemical Biology, College of Pharmacy and Tianjin Key Laboratory of Molecular Drug Research, Nankai University, Haihe Education Park, 38 Tongyan Road, Tianjin, 300353 People’s Republic of China; 2 Tianjin Key Laboratory of Molecular Drug Research, Tianjin International Joint Academy of Biomedicine, Tianjin, China

**Keywords:** Parthenolide, Pulmonary fibrosis, NF-κB/Snail signaling pathway

## Abstract

**Background:**

Parthenolide (PTL) is a natural molecule isolated from *Tanacetum parthenium* that exhibits excellent anti-inflammatory and antitumor activities. Pulmonary fibrosis (PF), especially idiopathic pulmonary fibrosis (IPF), is a chronic lung disease that lacks a proven effective therapy. The present study evaluated the therapeutic effect of PTL on PF.

**Methods:**

Serum-starved primary lung fibroblasts and HFL1 cells were treated with different doses of PTL, and cell viability and the migration rate were measured. Western blot analysis and a dual-luciferase assay were used to analyze the epithelial–mesenchymal transition (EMT)-related transcription factors influenced by PTL treatment in A549 cells and primary lung epithelial cells. Mice with bleomycin (BLM)-induced pulmonary fibrosis were treated with different doses of intragastric PTL, and pathological changes were evaluated using Hematoxylin-eosin (H&E) staining and immunohistochemical analysis.

**Results:**

Our results demonstrated that PTL reduced the cell viability and migration rate of lung fibroblasts and inhibited the expression of EMT-related transcription factors in lung epithelial cells. In vivo studies demonstrated that PTL attenuated BLM-induced pulmonary fibrosis and improved the body weight and pathological changes of BLM-treated mice. We further demonstrated that PTL attenuated BLM-induced PF primarily via inhibition of the NF-κB/Snail signaling pathway.

**Conclusion:**

These findings suggest that PTL inhibits EMT and attenuates BLM-induced PF via the NF-κB/Snail signaling pathway. PTL is a worthwhile candidate compound for pulmonary fibrosis therapy.

## Background

Pulmonary fibrosis (PF), especially idiopathic pulmonary fibrosis (IPF), is a chronic lung disease caused by several factors. IPF exhibits a complex pathogenesis, but no effective treatment is available for IPF. The mortality rate of IPF is considerably increased in recent years, and it substantially threatens human health [1, 2]. Current treatments for IPF, such as immunosuppressants (e.g., cyclophosphamide), are limited by low their efficacy and severe side effects. The FDA recently approved two new drugs, nintedanib and pirfenidone, to treat IPF. These drugs stabilize patients’ conditions well, but they do not reverse the progression of fibrosis. Both drugs produce side effects on the liver and skin, which limits their clinical application, especially in patients with liver problems [3, 4]. Recent research demonstrated that dexamethasone (DEX) attenuated bleomycin (BLM)-induced lung fibrosis [5]. However, DEX treatment produces many side effects, such as growth retardation, hyperglycemia, hypertension, myocardial hypertrophy, gastrointestinal perforation, and neurological impairment [6–8]. Therefore, new drugs with improved treatment efficacy and fewer side effects are urgently needed.

IPF is easily characterized by an excessive deposition of extracellular matrix (ECM), but the pathogenesis of IPF is not clear. Several hypotheses were proposed to explain the inner mechanisms, and epithelial–mesenchymal transition (EMT) of alveolar epithelial cells (AECs) received particular attention. Nuclear factor kappa-B (NF-κB) is an essential mediator of EMT. NF-κB promotes the transcription of many inflammatory cytokines, such as tumor necrosis factor α (TNF-α), interleukin (IL) and transforming growth factor β (TGF-β), which are highly associated with the progression of IPF, especially TGF-β [9–11]. Therefore, it is essential to measure these factors when evaluating the drug efficacy of IPF.

Parthenolide (PTL) is a sesquiterpene lactone that is isolated from the shoots of feverfew (*Tanacetum parthenium*), and PTL is a traditional medicinal herb used for headaches and arthritis. Recent studies suggested that PTL is a useful antitumor and anti-inflammatory agent, and it was tested in clinical studies for leukemia and neurological tumors [12]. These biological activities of PTL in tumor and inflammatory diseases primarily occur via inhibition of NF-κB and the targeting of multiple steps in the NF-κB signaling pathway. For example, PTL binds an activator of NF-κB, IκB-kinase (IKK) [13]. However, PTL treatment of IPF and its pharmacological properties have not been reported.

The present study found that PTL attenuated BLM-induced EMT-related protein expression and inhibited IPF-associated cytokines, which supports PTL as a potential compound for IPF treatment.

## Methods

### Reagents

PTL (> 99%) was provided by Shangdeyaoyuan Co. (Tianjin, China). DEX sodium phosphate (> 98.5%) was purchased from Meilun Biological Technology Co. (Dalian, China), and BLM sulfate (> 91%) was obtained from Meilun Biological Technology Co. (Dalian, China). The NF-κB, Snail, β-actin, GAPDH, E-cadherin, vimentin, MMP1, α-SMA and Col-1 antibodies were purchased from Affinity Biosciences Co. (Beijing, China). The mouse TNF-α, mouse IL-4, mouse TGF-β1, and mouse interferon gamma ELISA Kits were purchased from Meilian Biological Technology Co. (Shanghai, China). Chlorine ammonia T (> 97.08%) and *p*-dimethylaminobenzaldehyde (> 97.08%) were obtained from (> 99.71%). Reverse-4-hydroxy-l-proline (> 99.4%) was purchased from Bailingwei Technology Co. (Beijing, China). Perchloric acid (> 70%) was obtained from Jingchun Biological Technology Co. (Shanghai, China).

### Cell culture

The human pulmonary epithelial A549 cell line was obtained from KeyGen Biotech (Nanjing, China). The human fetal lung fibroblast cell line HFL1 was kindly supplied by Professor Wen Ning (Nankai University). The cells were cultured in a medium supplemented with 10% heat-inactivated (56 °C, 30 min) fetal calf serum (HyClone, USA) and maintained at 37 °C with 5% CO2 in a humidified atmosphere.

### Isolation of primary fibroblasts and AECs

Primary pulmonary fibroblasts isolated from NaCl/BLM-treated mice were cultured in DMEM supplemented with 10% FBS and antibiotics in 5% CO2 at 37 °C in a humidified atmosphere as described previously [14]. Cells at passages 3–4 were used for cell viability and wound healing assays. Primary AECs were isolated from C57BL/6 J mice as previously described [15]. Newly isolated AECs were used for immunofluorescence and Western blotting assays.

### Cell viability and wound-healing assays

Cell viability was determined using the MTT assay. Cells (5 × 10^3^ cells/mL) were seeded in 96-well culture plates and incubated overnight. Cells were treated with various concentrations of PTL for 24 h. Cell viability was measured after the addition of MTT (20 μL) at 37 °C for 4 h. Dimethyl sulfoxide (150 μL) was added to dissolve the formazan crystals. Optical density was measured at 570 nm using a microplate reader (Multiskan FC, Thermo Scientific, Waltham, MA, USA).

Cells for the wound healing assay were grown on a 35-mm dish to 100% confluency and scraped to form a 100-μm wound using sterile pipette tips. The cells were cultured in the presence or absence of PTL in serum-free media for 24 h. Images of the cells were obtained at 24 h using a light microscope (Nikon, Japan).

### Immunofluorescence

Primary epithelial cells were fixed in 4% paraformaldehyde for 20 min, washed with PBS, permeabilized with 0.2% Triton X-100 in PBS, blocked with 5% BSA and incubated with E-cadherin and vimentin antibodies. Cells were washed with PBS, and donkey anti-rabbit Fluor 555 or donkey anti-mouse Fluor 488 secondary antibodies (CWBIO, China) were used for immunofluorescence visualization. The nucleus was labeled with DAPI (Solarbio, China), and cells were photographed with a TCS SP5 confocal (Leica) microscope.

### Dual luciferase assay

AP1, STAT3, NF-κB, snail, slug and MYC promoters were cloned into the pGL6-TA luciferase reporter vector, and A549 cells were transfected with luciferase reporter plasmids using Lipofectamine (Invitrogen). Renilla-luciferase was used as an internal control. Cells were treated 1 d after transfection with 5 μΜ (L) or 10 μM (H) PTL for 24 h. Cells were harvested, and the luciferase activity of cell lysates was determined using a luciferase assay system (Promega) as described by the manufacturer. Total light emission was measured using a Luminoskan Ascent Reader System (Thermo, Massachusetts, USA).

### BLM-induced PF in mice

Specific pathogen-free ICR mice (males) (body weights 18–22 g) were purchased from the Laboratory Animal Center, Academy of Military Medical Sciences of People’s Liberation Army (Beijing, China) and housed in groups of six under a regular 12-h light/dark cycle. Mice were acclimated to laboratory conditions for one week prior to testing at a constant temperature.

Sixty mice were divided into six groups with 10 animals per group according to body weight: control group, BLM group, BLM + DEX group (0.45 mg/kg), BLM + PTL-H group (50 mg/kg), BLM + PTL-M group (25 mg/kg), and BLM + PTL-L group (12.5 mg/kg). PF was established in mice via a single intratracheal administration of BLM at 5 mg/kg body weight. Different doses of PTL were intragastrically administered daily for four weeks beginning 7 days after BLM injury, and DEX was used as the positive control. Control and model groups received an equal volume of vehicle (0.9% NaCl) using the same schedule and route of administration.

Mouse body weights were recorded daily. Mice were sacrificed on the 36th day using excess chloral hydrate hydrochloride anesthesia. Blood was obtained for ELISA analyses, and whole lungs were removed and weighed. The right lungs were fixed in 10% formalin, dehydrated, and embedded in paraffin. The left lungs were used to determine hydroxyproline. The pulmonary coefficient was calculated using the following equation: lung weight/body weight × 100%.

## Hydroxyproline assay

Collagen contents in left lungs of each group were measured using a conventional hydroxyproline method [15]. The results were confirmed via measurement of samples containing known amounts of purified collagen.

### Evaluation of pulmonary function

Mice were anesthetized with 10% chloral hydrate in NaCl (i.p.) and transferred to a plethysmographic chamber for pulmonary function analyses using the Anires2005 system (Beijing Biolab, Beijing, China). This system automatically calculates and displays pulmonary function parameters, including dynamic compliance and inspiratory and expiratory resistance.

### Histopathological examination

Paraffin sections were prepared at a 4-μm thickness, stained with H&E and Masson’s trichrome using the manufacturers’ standard procedures, and observed under a photomicroscope (Olympus, Tokyo, Japan) for microscopic examination of morphological changes and fibrosis evaluation (collagen fibers).

### Immunohistochemistry

The tissue sections were pretreated in a microwave, blocked and incubated using a series of antibodies, and stained with DAB and hematoxylin. The results were captured using a microscope (Olympus, Japan). The intensity and percentage of positive cells were measured. Multiplication (staining index) of intensity and percentage scores was used to determine the results.

### Plasma collection

Mice were anesthetized, and a microhematocrit tube was introduced to the canthus of the orbit. The microhematocrit tube was slightly advanced and rotated to allow blood flow into the lithium-heparin tube. Plasma was separated from the cellular fraction via centrifugation at 3500 rpm for 10 min at 4 °C and stored at − 80 °C.

### Bronchoalveolar lavage fluid (BALF) collection and cell counts

The tracheas of mice were cannulated and lavaged three times with 1-ml sterile PBS at room temperature for BALF collection. Samples were centrifuged at 1000 rpm for 5 min, and cell pellets were recovered in 1-ml sterile PBS. Cells were counted using a hemocytometer. Smears of BALF cells were stained with hematoxylin and eosin and viewed under light microscopy to measure the inflammatory cell differential.

### TGF-β1, TNF-α and IL-4 assays

Plasma TGF-β1, TNF-α and IL-4 levels were assayed using ELISA Kits (Shanghai Enzyme-linked Biotechnology Co., Ltd., Shanghai, China). Assays were performed according to the manufacturer’s instructions.

### Statistical analysis

Data are presented as the means ± standard deviation. Significant differences between treatment groups were detected using one-way ANOVA. All analyses were performed using SPSS 17.0 statistical software. *P* < 0.05 was considered statistically significant.

## Results

### PTL reduces cell viability and inhibits the migration of lung fibroblasts

**Fig. 1:**
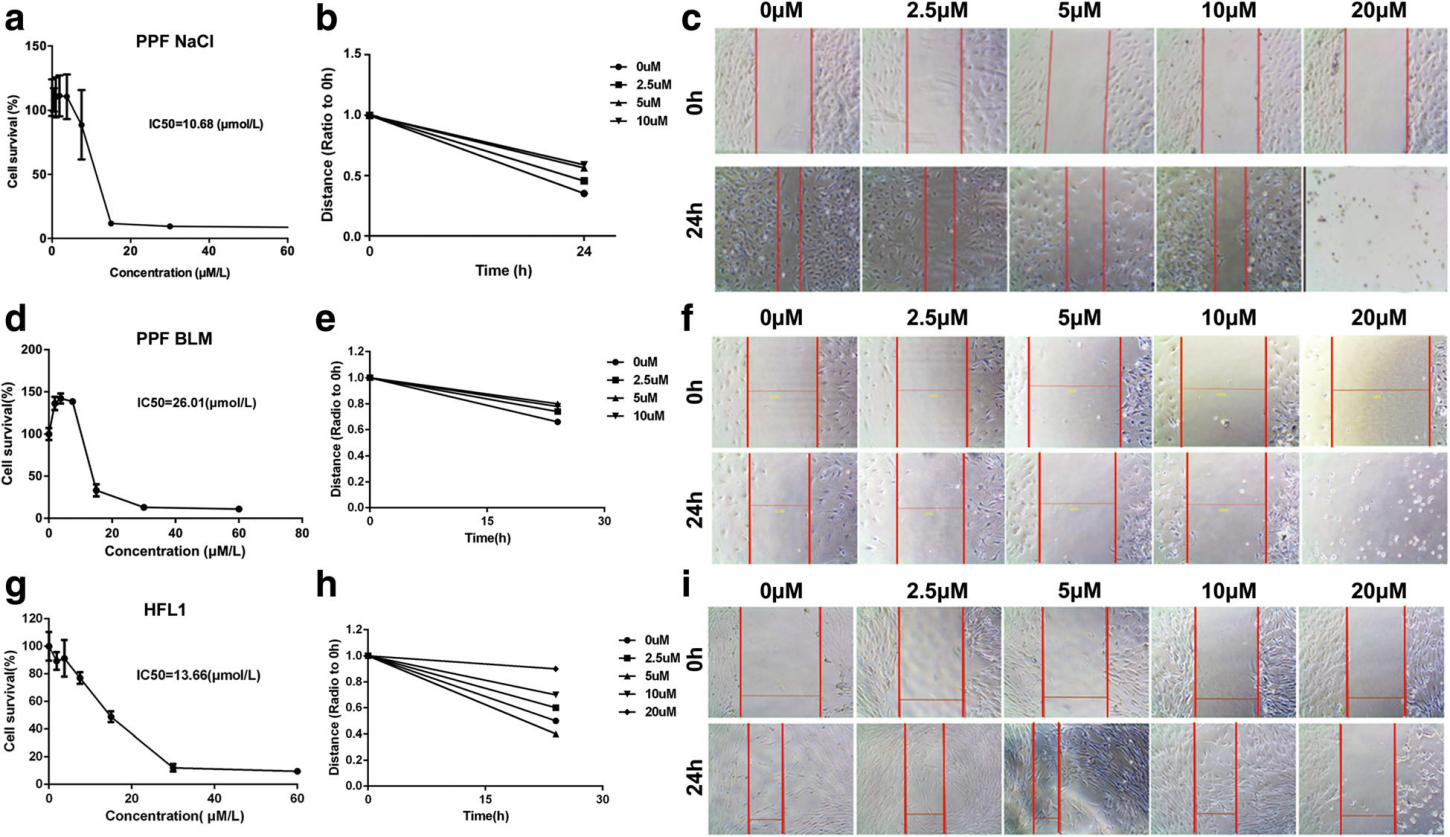
PTL reduces cell viability and migration of primary pulmonary fibroblasts (PPF) and HFL1 cells (**a**) The PPF isolated from NaCl-treated mice were treated with indicated dosages of PTL for 24 h (IC_50_ = 10.68 μM). PTL reduced the cell viability in a dose-dependent manner. **b**, **c** The PPF isolated from NaCl-treated mice were incubated in a medium containing PTL (0, 2.5, 5, 10, and 20 μM) for 24 h. Results showed that cell viability and migration were inhibited after incubation in different groups for 24 h. **d** The PPF isolated from BLM-treated mice were treated with indicated dosages of PTL for 24 h (IC_50_ = 26.01 μM). PTL reduced the cell viability in a dose-dependent manner. **e**, **f** The PPF isolated from BLM-treated mice were incubated in a medium containing PTL (0, 2.5, 5, 10, and 20 μM) for 24 h. **g** The HFL1 cells were treated with indicated dosages of PTL for 24 h (IC_50_ = 13.66 μM). PTL reduced the cell viability in a dose-dependent manner. **h**, **i** The HFL1 cells were incubated in a medium containing PTL (0, 2.5, 5, 10, and 20 μM) for 24 h

We determined the effect of PTL treatment (24 h) on the cell viability of primary pulmonary fibroblasts (PPF) isolated from NaCl/BLM-treated mice and HFL1 cell lines using MTT assays (Fig. 1a, d and g). The half-maximal inhibitory concentrations (IC_50_) of PTL after 24-h treatment in the PPF-NaCl, PPF-BLM and HFL1 cells were 10.68 μM, 26.01 μM and 13.66 μM, respectively. These results indicate that PTL reduced the cell viability of lung fibroblasts in a dose-dependent manner.

The effect of PTL on the cellular migration of lung fibroblasts was determined using a wound-healing assay. Confluent cells were scraped with a sterile pipette tip, and the remaining cells were allowed to migrate to the resulting gap in the absence or presence of PTL. Serum-starved PPF or HFL1 cells in the control group exhibited a narrow cell wound gap in the wound area 24 h after wounding. Cells treated with PTL (2.5, 5, 10, or 20 μM) exhibited relative delays in wound closure (Fig. 1b-c, e-f and h-i).

### PTL inhibits the expression of EMT-related transcription factors

**Fig. 2:**
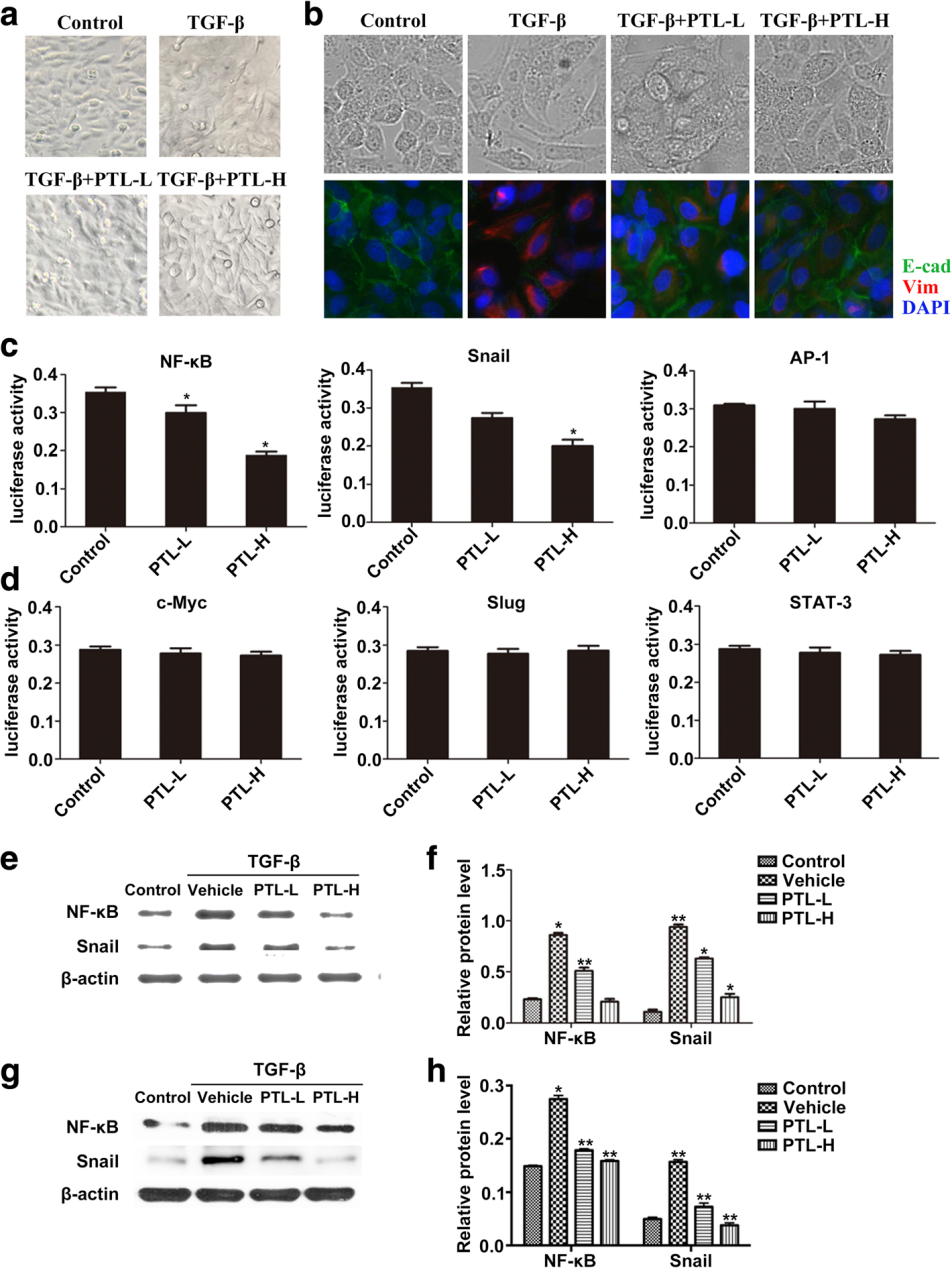
PTL inhibits TGF-β1-induced EMT through inhibiting NF-κB/Snail expression in lung epithelial cells. **a** Typical images of A549 cells in the Control group, TGF-β group, PTL-L + TGF-β group, and PTL-H + TGF-β group under an optical microscope. **b** Typical images of primary lung epithelial cells in the Control group, TGF-β group, PTL-L + TGF-β group, and PTL-H + TGF-β group under an optical microscope and immunofuorescence staining of E-Cadherin (green) and Vimentin (red) was performed. The nucleus was staining with DAPI. **c**, **d** Expression levels of NF-κB, Snail, AP-1, c-Myc, Slug and Stat-3 were assessed using dual-luciferase assay. **e**-**h** After TGF-β/PTL treatment, NF-κB and Snail were evaluated using Western blot analysis. β-actin was used as a loading control. Data are presented as means of three experiments; error bars represent standard deviation, *P < 0.05, **P < 0.01

We researched the influence of PTL on the TGF-β1-induced EMT process in lung epithelial cells to further investigate its effects on the pathological mechanisms of PF. Figure 2a shows that low doses of TGF-β, an inducer of EMT, resulted in extended pseudopodia and changes in the microfilament structure of A549 cells. The PTL-H + TGF-β and PTL-L + TGF-β groups reversed the features of EMT to different degrees. The morphology changes and EMT marker expression in TGF-β/PTL-treated primary AECs exhibited the same results (Fig. 2b). We further evaluated several EMT-related transcription factors, such as NF-κB, Snail, AP-1, c-Myc, Slug, and Stat-3. We analyzed the activity of these transcription factors in A549 cells using a dual-luciferase assay. PTL reduced the expression and activity of NF-κB and Snail, but did not influence the expression of AP-1, c-Myc, Slug, and Stat-3 (Fig. 2c and d).

We verified the effect of PTL treatment on the expression of EMT-related transcription factors NF-κB and Snail in A549 cells and primary AECs. Western blot analysis results revealed that NF-κB and Snail levels increased significantly in the A549 cells and primary AECs after exposure to TGF-β. NF-κB and Snail levels decreased slightly in the TGF-β + PTL-L group and decreased severely in the TGF-β + PTL-H group (Fig. 2e-h). The expression levels of Snail and NF-κB decreased in a dose-dependent manner.

### PTL attenuates the BLM-induced PF in mice

**Fig. 3:**
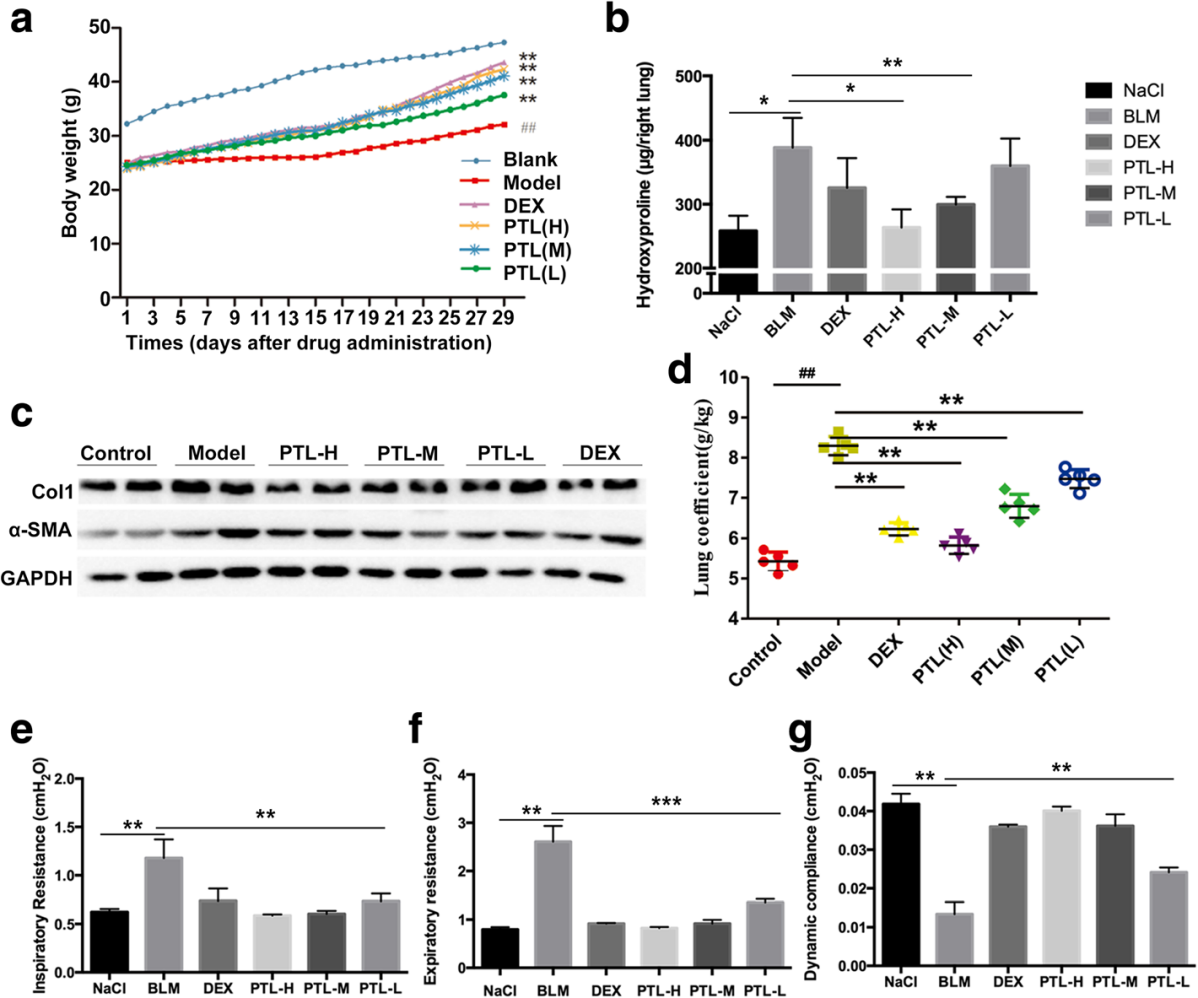
PTL attenuates the BLM-induced pulmonary fibrosis in mice. **a** Body weights (g) of animals after PTL treatment. **b** Hydroxyproline content in lung tissues. **c** Western blot analysis of type I collagen (Col1) and α-SMA in lung tissues was performed. GAPDH was used as a loading control. **d** Lung coefficient levels in the mouse model of BLM-induced PF. **e**-**g** Pulmonary function parameters including inspiratory resistance, expiratory resistance and pulmonary dynamic compliance among different groups were compared. Data are expressed as mean ± SD, *P < 0.05, **P < 0.01,***P < 0.001

Mice were intragastrically administered with or without PTL after BLM injection to investigate the effects of PTL on BLM-induced lung fibrosis. A protective effect of PTL was observed on weight loss. Body weight loss was significantly attenuated in mice treated with PTL compared to the model group (Fig. 3a). Treatment of bleomycin-injured mice with high/middle PTL doses significantly reduced collagen content compared to mice treated with DEX (Fig. 3b). The protein levels of collagen (Col1) and α-SMA exhibited similar results (Fig. 3c). The lung coefficients were higher in the lung tissues of the model group than the other groups, and the lung coefficient of the mice treated with PTL was considerably reduced compared to the model group (Fig. 3d). The attenuated fibrosis in PTL-treated mice was further supported by improved pulmonary function, which was observed as a decreased inspiratory resistance, expiratory resistance and increased dynamic compliance compared to BLM-treated mice (Fig. 3e-g). Collectively, these in vivo data indicate that PTL attenuated bleomycin-induced pulmonary fibrosis in mice.

### PTL inhibits the inflammatory responses of PF

**Fig. 4:**
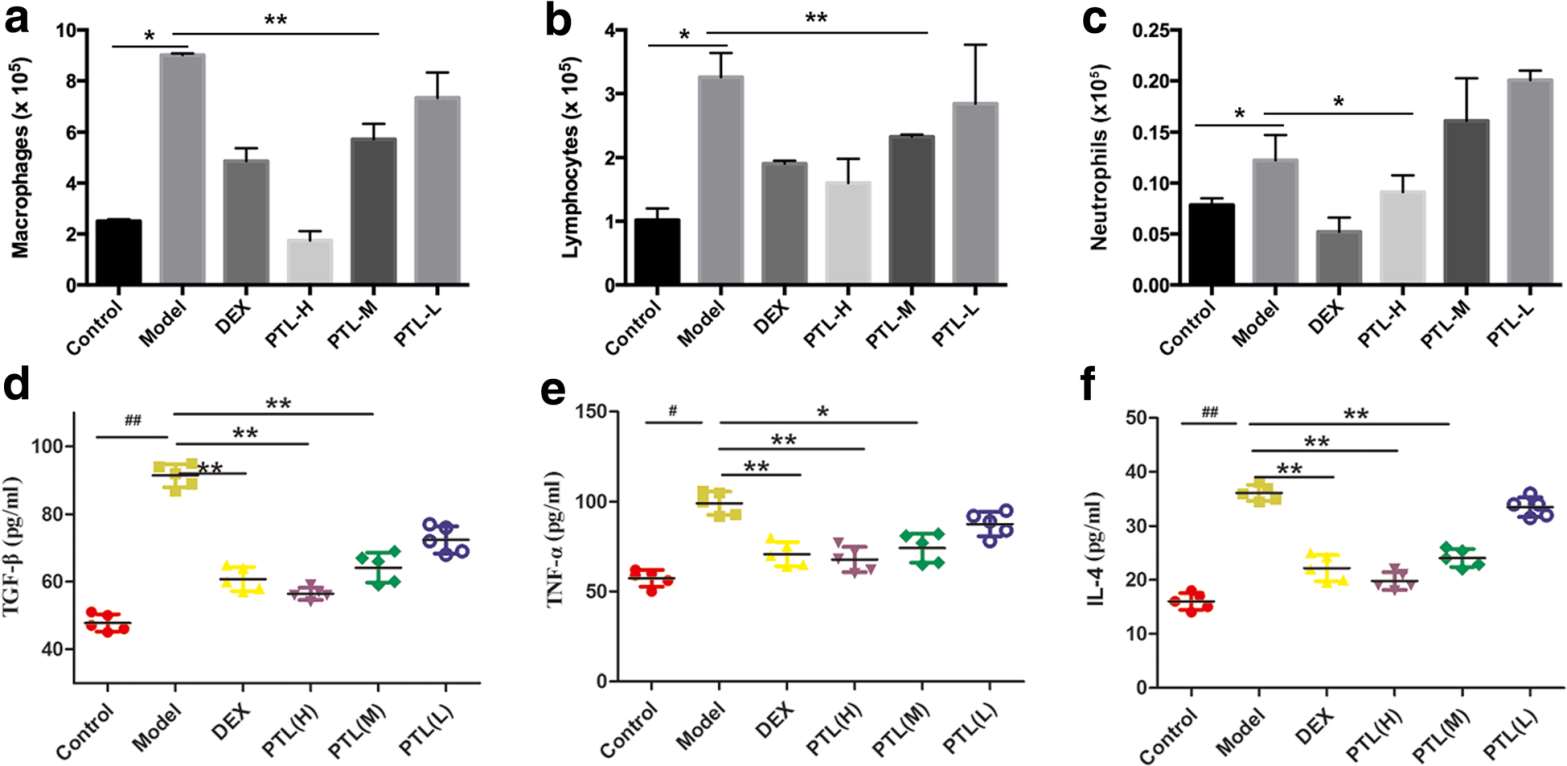
PTL attenuates BLM-induced lung inflammatory responses. **a**-**c** The different inflammatory cell counts in BALF (**a**: Macrophages, **b**: Lymphocytes, **c**: Neutrophils) were determined by standard morphologic criteria; (**d**-**f**) The expression levels of TGF-β, TNF-α and IL-4 in plasma of each group were evaluated with ELISA assay. All data are presented as means of three experiments; error bars represent standard deviation (*P < 0.05, **P < 0.01)

Persistent inflammation exists in an early stage, and it drives fibrotic progression in bleomycin-induced fibrosis. However, the effect of inflammation in fibrogenesis is arguable. Inflammatory cell numbers in BALF and inflammatory cytokines in plasma were evaluated to determine whether PTL altered inflammatory responses. Notably, cell differentiation analysis of BALF revealed that the increase in macrophages, lymphocytes and neutrophils was attenuated in the high/middle PTL dose groups compared to the model group (Fig. 4a-c). TGF-β, TNF-α and IL-4 levels in plasma were detected using ELISA. Levels of the inflammatory cytokines TGF-β, TNF-α and IL-4 were increased in the plasma of the model group compared to the other groups. The levels of these inflammatory cytokines decreased significantly in mice treated with PTL compared to untreated mice, and this effect was dose-dependent. Our assay results demonstrated that PTL significantly reduced the inflammatory response in bleomycin-injured mice in a dose-dependent manner.

### Effects of PTL on histopathological changes in vivo

**Fig. 5:**
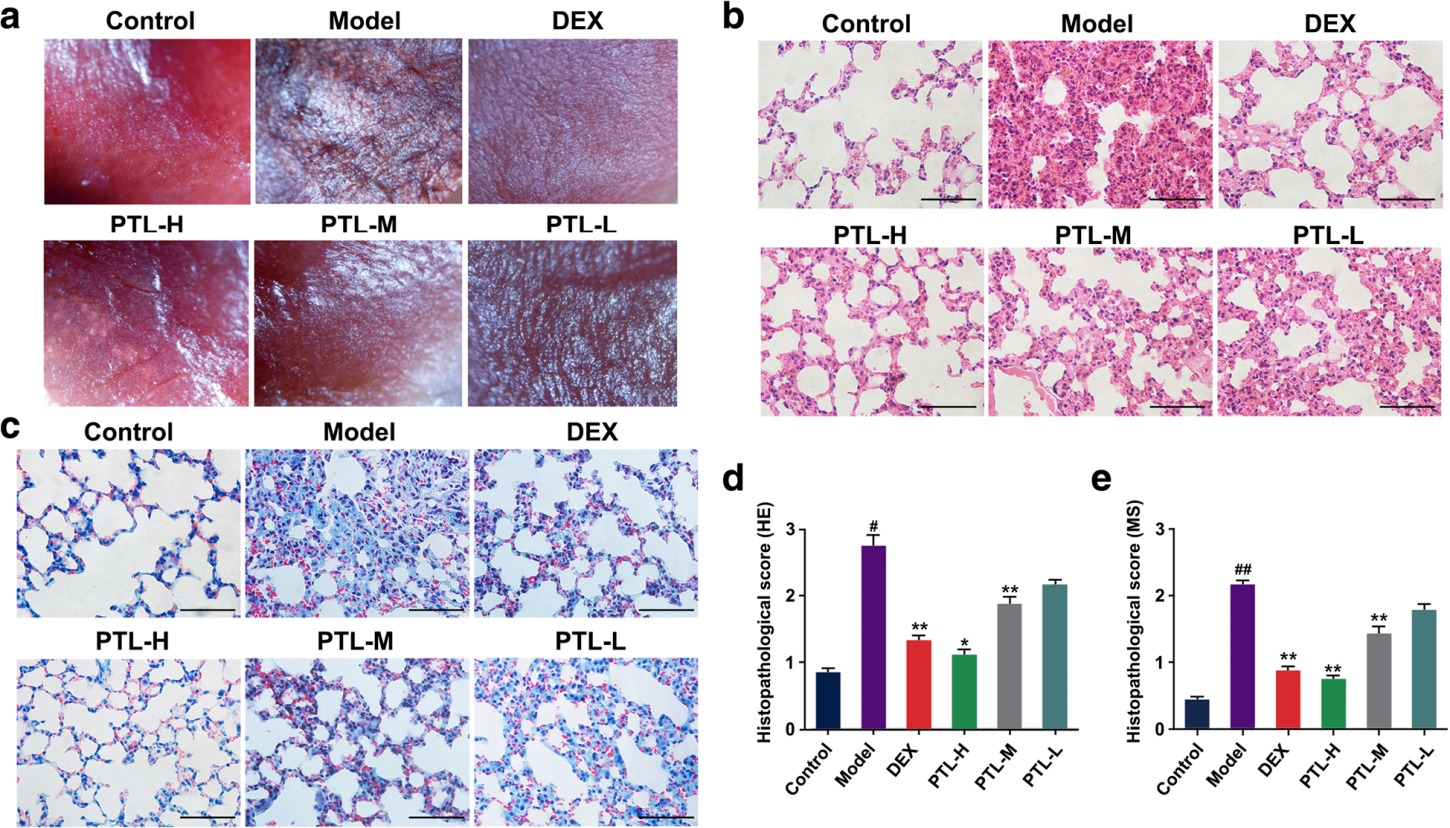
Effects of PTL on histopathological change in vivo. **a** Gross appearance of the lungs under the stereoscopic microscope. **b**, **d** Photomicrographs of lung sections stained with hematoxylin–eosin (HE). **c**, **e** Masson trichrome staining of collagen on lung sections. (*P < 0.05, **P < 0.01)

Lung tissues were pink and soft under the normal conditions, and the lungs of mice in the bleomycin-treated model group were hard, shrunken in size, and dark in color. Treatment with PTL remarkably improved the lung condition (Fig. 5a). Histopathological changes in mouse lung tissues were evaluated using H&E staining. The lung tissue specimens of the BLM-induced mice treated with PTL exhibited marked improvements in inflammation and fibrosis (Fig. 5b and c). The BLM-injured lungs exhibited fibroplasia, inflammatory cell infiltration, thickened alveolar walls, destroyed and disordered alveoli, and stenosed or partially collapsed alveolar spaces (Fig. 5b and d). The lungs of the animals treated with PTL revealed significantly reduced infiltration of inflammatory cells, edema, thrombosis, and structure destruction compared to the model group. Masson’s trichrome staining was used to examine the collagen deposition and distribution and assess fibrosis in lung tissues. Significant collagen deposition, particularly around the bronchus, was observed in BLM-injured lung tissues compared to their respective controls (Fig. 5c and e). The PTL-treated groups exhibited a significant decrease in collagen deposition compared to the model group, and PTL-H was better than PTL-L.

### PTL attenuates the BLM-induced EMT-related protein expression and inhibits cytokine production of PF

**Fig. 6:**
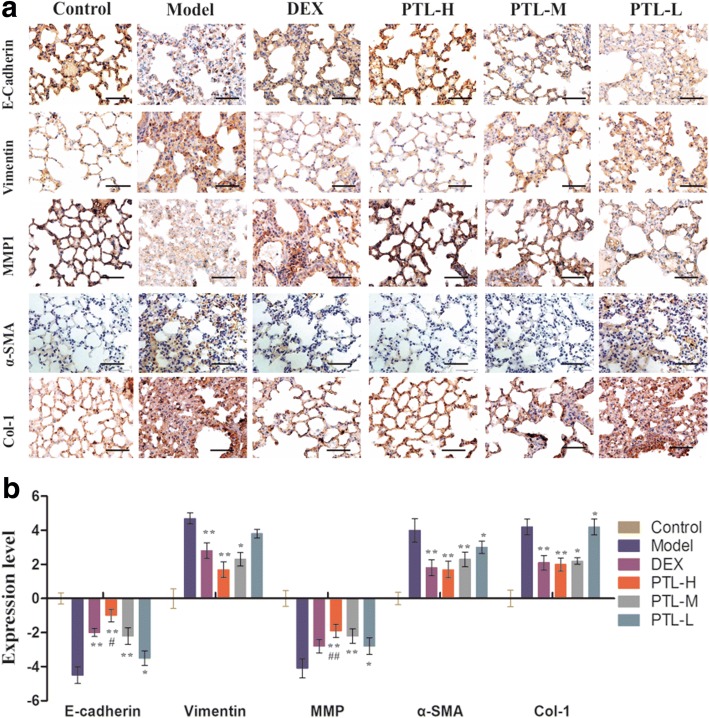
PTL attenuates the BLM-induced expression of EMT-related protein. **a** Representative immunohistochemical staining of lung sections showing E-cadherin, vimentin, MMP1, α-SMA and Col-1 staining. **b** Expression levels of makers were evaluated by index of immunohistochemical staining. The number of positively stained cells in each group was calculated from twenty different 400× magnified fields under a microscope. The data represent the mean ± standard deviation (SD), *n* = 10 per group. *, *P* < 0.05, **P < 0.01 vs. model group

The present study analyzed several EMT-related proteins to determine whether PTL affected the conversion of AECs to fibroblasts (FBs). PTL increased epithelial cell markers, such as E-cad, and reduced the vimentin and α-SMA (marker of mesenchymal cells) expression (Fig. 6). These results suggest that PTL increased epithelial cell markers and reduced mesenchymal cell markers. The effect of PTL was better than DEX. PTL also inhibits Col-1 and MMP1, which are PF cytokines. Immunohistochemical staining of Col-1 and MMP1 revealed that PTL increased MMP1 levels and decreased Col-1 levels in lung tissues in a dose-dependent manner (Fig. 6).

**Fig. 7:**
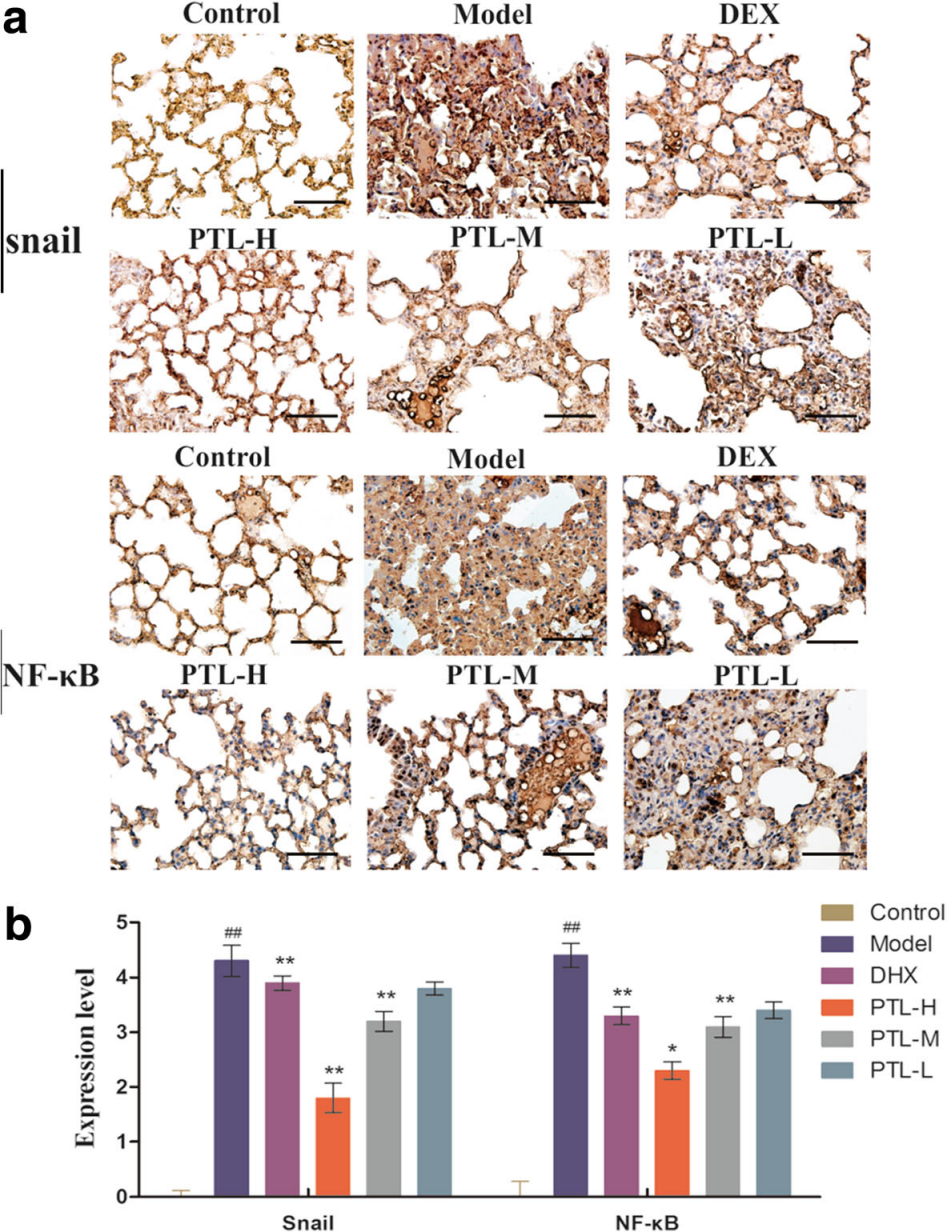
PTL attenuates the BLM-induced expression of NF-κB and Snail. **a** Immunohistochemical staining for NF-κB and Snail in lung sections. **b** Expression levels of NF-κB and Snail were evaluated by index of immunohistochemical staining. The number of positively stained cells was calculated from twenty different 400× magnified fields under a microscope. The data represent the mean ± standard deviation (SD), n = 10 per group. *, *P* < 0.05, **P < 0.01 vs. model group

The expression levels of NF-κB and Snail were also assessed using immunohistochemical staining. The results demonstrated that NF-κB and Snail levels decreased significantly compared to the model group in a dose-dependent manner (Fig. 7). PTL inhibited EMT via the NF-κB/Snail pathway in lung tissues. These results are consistent with the cell experimental results.

### Mechanism research on PTL and large data analysis using online database

**Fig. 8:**
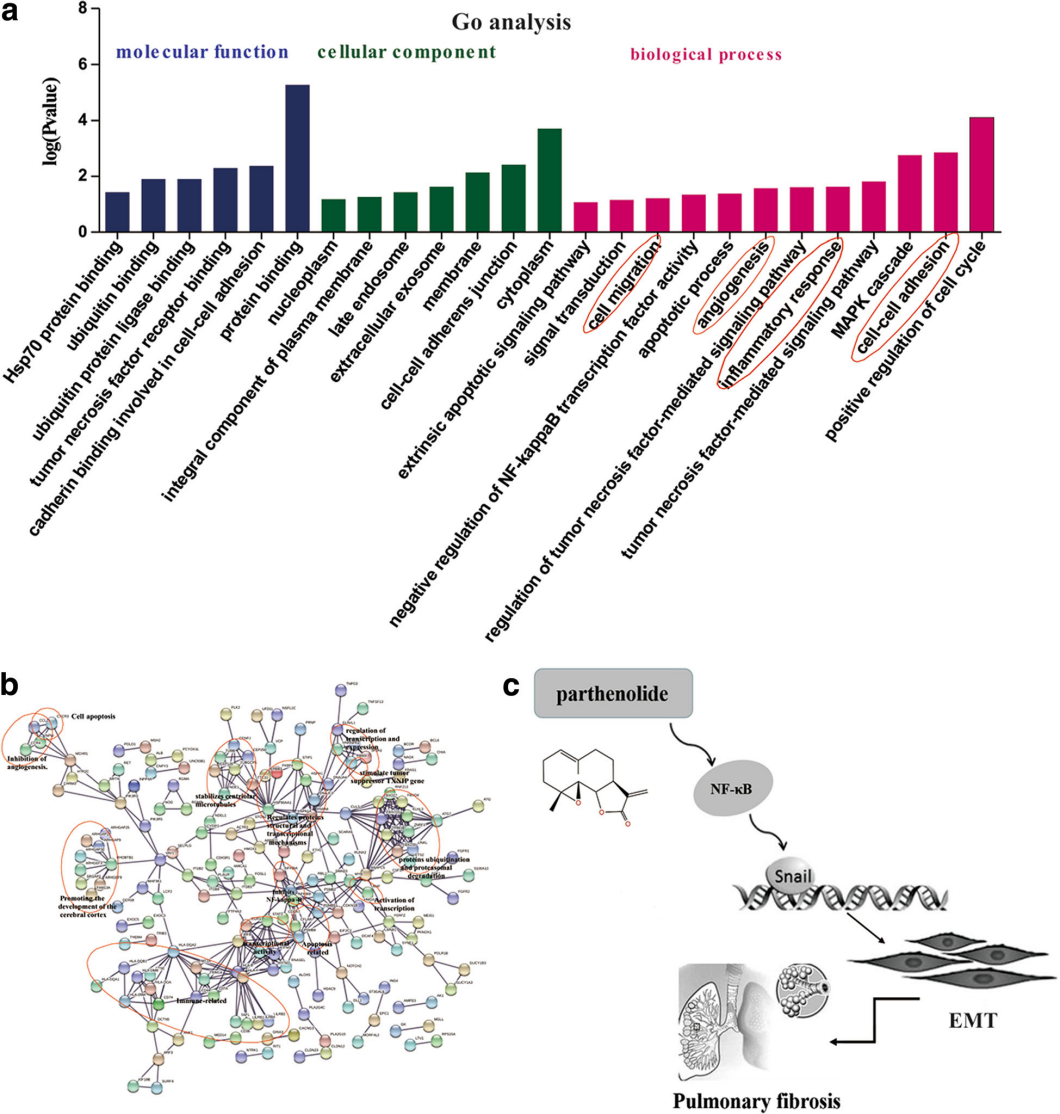
Mechanism research on the PTL effects. **a-b** GO analysis of PTL in affecting biological processes. **c** A model showing the role of PTL in BLM-induced EMT

The STRING database was used to examine several types of interactions between the control and PTL treatment groups. Gene Ontology (GO) analysis was performed to analyze the function of differentially expressed mRNAs using the GO categories. GO categories are derived from Gene Ontology (http://www.geneontology.org), and the categories include three integrated networks of defined terms that describe gene properties (molecular function, cellular component and biological process). PTL influenced many functions, including inflammatory responses and proliferation (Fig. 8a). We also analyzed the molecular function, cell structure, and biological processes of the expression profiling chip on the GO website after PTL treatment and compared the results with the control groups (Fig. 8b). These biological processes are closely related to pulmonary fibrosis.

## Discussion

PTL is a natural molecule that was originally isolated from the shoots of feverfew (*Tanacetum parthenium*). PTL exhibits excellent anti-inflammatory and antitumor activities [16, 17]. The first written records of the anti-inflammatory effect of PTL were provided in 1597 in Europe [18]. PTL from *Magnolia grandiflora* exhibited antitumor properties for the first time in 1973 [19]. The biological properties of PTL were primarily attributed to the strong inhibition of NF-κB and the targeting of various steps within the NF-κB signaling pathway [20, 21].

The inflammatory response during the initial phase of PF damages the ECM and produces numerous FBs via activation of the repair mechanism. The relationship between PF and cytokines, particularly TNF-α and TGF-β, attracted considerable attention in recent years. These cytokines promote inflammation progression [22–24]. NF-κB is commonly activated to protect against organisms, but disorder in its activation is related to chronic inflammation [25]. PTL inhibited the activities of TNF-α, TGF-β, and NF-κB in the present study.

Our studies evaluated the role of PTL in PF. The results demonstrated that PTL repressed BLM-induced pulmonary fibrosis in mice. PTL inhibited EMT of AECs, which upregulate epithelial marker expression and downregulate the expression of mesenchymal markers. We evaluated the influence of PTL on AP-1, NF-κB, STAT-3, Snail, Slug and c-Myc expression. PTL only downregulated NF-κB and Snail. The NF-κB pathway regulates Snail expression via transcriptional and post-translational mechanisms. NF-κB binds to the human Snail promoter and increases Snail transcription. Our results demonstrated that NF-κB and Snail expression levels and activities decreased following PTL treatment. Therefore, PTL may inhibit the NF-κB signaling pathway and exhibit proinflammatory effects during PF.

FBs are important in the structural formation process and maintaining the function of pulmonary tissues [26]. The cross talk of FBs and AECs promotes fibrosis. FBs proliferate continuously as a result of multiple factors, such as the stimulating action of cytokines [27–29]. Therefore, an effective approach to inhibit FB proliferation in PF should be urgently identified [30]. The present results demonstrated that PTL inhibited the proliferation and migration of primary pulmonary fibroblasts and HFL1 cells in a dose-dependent manner.

EMT is an indispensable step in numerous diseases, and it induces cell changes involved in pathological processes, such as fibrosis [31, 32]. EMT plays a pivotal role in the development of PF. Lung epithelial cells are a frequent target of injury, a driver of normal repair, and a key element in the pathobiology of fibrotic lung diseases. One important aspect of epithelial cells is their capacity to respond to microenvironmental cues by undergoing EMT. EMT regulates a series of critical signaling elements that produce proinflammatory signals and cause cell injury. EMT is not the widespread conversion of epithelial cells to FBs, but it is a graded response whereby epithelial cells reversibly acquire mesenchymal features and enhance the capacity for mesenchymal cross talk [33]. Repeated injury superimposes persistent inflammation and hypoxia in these highly regulated repair pathways, which potentially overwhelms the orderly repair to create sustained fibrogenesis [34]. Our results suggest that PTL inhibited PF via inhibition of EMT. PTL increased the expression level of E-cad and reduced vimentin levels in TGF-β1-treated primary lung epithelial cells. NF-κB and Snail levels decreased significantly in the PTL treatment groups in a dose-dependent manner.

The potential signaling pathways involved in PTL treatment were analyzed using the STRING database and GO analysis. PTL affected many functions, including the inflammatory response, proliferation, molecular function, cell structure, and biological processes. These biological processes were closely related to pulmonary fibrosis.

## Conclusion

PTL significantly ameliorated BLM-induced lung fibrosis via the NF-κB/Snail signaling pathway and inhibited EMT (Fig. 8c). PTL may be a worthwhile candidate compound for pulmonary fibrosis therapy.

## Abbreviations

AECs: Alveolar epithelial cells
BALF: Bronchoalveolar lavage fluid
BLM: Bleomycin
DEX: Dexamethasone
ECM: Extracellular matrix
EMT: Epithelial–mesenchymal transition
FBs: Fibroblasts
FDA: Food and drug administration
H&E: Hematoxylin-eosin
IL: Interleukin
IPF: Idiopathic pulmonary fibrosis
MMP: Matrix metalloproteinase
PF: Pulmonary fibrosis
PPF: Primary pulmonary fibroblasts
PTL: Parthenolide
TGF-β: Transforming growth factor β
TNF-α: Tumor necrosis factor α
α-SMA: α-smooth muscle actin
